# Thirty years of HIV pregnancies in French Guiana: prevention successes and remaining obstetrical challenges

**DOI:** 10.3389/fgwh.2023.1264837

**Published:** 2024-01-03

**Authors:** Mathieu Nacher, Julie Blanc, Sebastien Rabier, Aude Lucarelli, Antoine Adenis, Celia Basurko, Alphonse Louis, Dominique Dotou, Malika Leneuve, Lindsay Osei, Narcisse Elenga, Najeh Hcini

**Affiliations:** ^1^ Centre d'Investigation Clinique INSERM 1424, Centre Hospitalier de Cayenne, Cayenne, French Guiana; ^2^ Amazonian Infrastructures for Population Health, Centre Hospitalier de Cayenne, Cayenne, French Guiana; ^3^ Département Formation Recherche Santé, Université de Guyane, Cayenne, French Guiana; ^4^ COREVIH Guyane, Centre Hospitalier de Cayenne, Cayenne, French Guiana; ^5^ Service de Gynécologie Obstétrique, Centre Hospitalier de Cayenne, Cayenne, French Guiana; ^6^ Collectivité territoriale de Guyane, Protection Maternelle et Infantile, Cayenne, French Guiana; ^7^ Service de Pédiatrie, Centre Hospitalier de Cayenne, Cayenne, French Guiana; ^8^ Service de Gynécologie Obstétrique, Centre Hospitalier de l’Ouest Guyanais, Saint Laurent du Maroni, French Guiana

**Keywords:** HIV, pregnancy, vertical transmission, preterm delivery, French Guiana

## Abstract

**Introduction:**

In a context of high HIV prevalence, poor pregnancy follow-up, frequent poverty, preeclampsia, and preterm delivery, we aimed to describe the characteristics and outcomes of pregnancies among women living with HIV in French Guiana.

**Methods:**

A retrospective cohort study was conducted on HIV-infected pregnancies enrolled between January 1st 1992 to 31st July 2022. Overall, there were 1,774 pregnancies in 881 women living with HIV.

**Results:**

For 75.1% of pregnancies, the HIV diagnosis was already known before pregnancy and in 67.6% of women, HIV follow-up predated pregnancy. Nearly half of women, 49.6%, only had one pregnancy since having been diagnosed with HIV. Although most women received antiretroviral therapy during pregnancy, for those with the available information we found only 48.5% had an undetectable viral load at delivery. Overall, 15.3% of pregnancies ended with an abortion. There were a total of 110 newborns infected with HIV representing an overall transmission rate of 6.2% (110/1,771). Between 1993 and 2002, the transmission rate was 34%, between 2003 and 2012 it was 1.3%, and between 2013 and 2022 it was 0.7%. Overall, in Cayenne, since 2008, 106 of 581 HIV–infected pregnancies (18.2%) with available information were premature before 37 weeks of pregnancy; of these, 33 (5.7%) were very preterm deliveries and 73 (13.3%) were late preterm deliveries. Over time, in Cayenne, preterm delivery declined significantly.

**Conclusions:**

The present study emphasizes that, despite spectacular progress in reducing mother to child transmission, pregnancy outcomes among women living with HIV are still preoccupying with high incidence of preterm delivery and low birth weight. Teasing out what fraction is linked to HIV and what fraction is linked to social precariousness and poor follow-up was not possible in this study. Despite the high incidence of very preterm delivery recent progress suggests that coordination efforts to improve follow-up may also have improved obstetrical outcomes.

## Introduction

French Guiana is a French territory in South America that shares borders with Brazil and Suriname. For cultural reasons, it has the highest birth rate in Latin America (26.8 per 1,000 persons) (1). The HIV prevalence and incidence are the highest of all French territories. The prevalence of HIV in French Guiana in the 15–49 year-old age group is estimated at 1.6% and incidence has substantially declined since the advent of highly active antiretroviral therapy (2). The drivers of the epidemic are: frequent multiple sexual partnerships, transactional sex, and crack cocaine-related risky sex behaviors (3). These drivers are often related to social precariousness. Because French Guiana has the highest Gross Domestic Product (GDP) and health expenditure per capita in Latin America, it attracts numerous vulnerable immigrants who arrive in hope of better economic prospects. However, when they arrive, immigrants often live in great poverty. While 29% of the total population and nearly half of adults are foreigners, for persons living with the Human Immunodeficiency Virus (HIV), 80% are foreigners (3). Although many immigrants with HIV come from HIV endemic countries, we have shown that over half acquire the infection early after arrival in French Guiana, presumably because of enhanced sexual vulnerability linked to social precariousness (4, 5). In this context, the transmission mode of HIV is by far mostly heterosexual, with a balanced number of males and females. The health system is the French universal health care system, which provides mostly free health care to all (genotyping, viral load and CD4 lymphocyte measurements, and antiretrovirals, among others), including undocumented immigrants, most of whom benefit from residence permits for health reasons. However, for persons who have a difficult life, access to care is often complicated or secondary to the immediate pressing needs of survival. This is particularly obvious for pregnant women, over a third of whom have a poor pregnancy follow-up (6). Preterm delivery is double that of mainland France (13% vs. 6.9%, respectively) (7). Stillbirths and infant mortality are both 2–3 times greater than in France (8). The prevalence of HIV among pregnant women in French Guiana has exceeded 1% for over 3 decades. Since 2008, following the national French guidelines, all pregnant women receive a triple antiretroviral combination therapy. HIV during pregnancy has been associated with a number of adverse outcomes (9–12). The role of antiretrovirals has yielded contrasting results: some finding no difference in pregnancy outcomes (13) whereas others, notably a case control study in French Guiana, found antiretroviral treatment was associated with preterm delivery (12).

In this context of high HIV prevalence, frequent poverty, poor pregnancy follow-up, frequent preeclampsia, frequent preterm delivery, we hypothesized that describing pregnancy outcomes and their trends would provide precious information to optimize the care of women with HIV. We thus aimed to describe the characteristics and outcomes of 1,774 pregnancies among a historical hospital cohort of 881 women living with HIV in French Guiana. The Amazonian context of the study may provide useful information for other countries in the region.

## Methods

The study was a retrospective cohort conducted on the French Guiana data included in the French Hospital Database for HIV (FHDH), a national cohort that prospectively records all events and characteristics of persons with HIV. HIV-infected pregnancies (confirmed infections by western blot analysis) enrolled between January 1st 1992 to 31st July 2022 were considered. While HIV was the exposure the main outcomes of interest were pregnancy outcome, transmission, obstetrical complications. Patient data are routinely entered in the database by physicians as they consult and trained research technicians who control for exhaustivity. The main study variables were age, country of birth, date of HIV discovery and reason for testing, initial CD4 count, CDC stage, viral load, antiretroviral treatment, opportunistic infections, and pregnancy outcomes (obstetrical complications, labor and delivery complications, early neonatal outcomes, complications related to puerperium). Because HIV records did not have precise information about term at delivery, between 2008 and 2022 research technicians compiled this information in Cayenne.

### Statistical analysis

Descriptive analysis computed means and standard deviations for quantitative variables, and counts and percentages for qualitative and ordinal variables. Pregnancy complications were studied using International Classification of Diseases (ICD 10) codes O10–O16 (edema, proteinuria, and hypertensive disorders in pregnancy, childbirth, and the puerperium), O20–O29 (Other maternal disorders predominantly related to pregnancy), O40–O48 (Ectopic pregnancy, molar pregnancy, and other abnormal conceptions), O60–O75 (Complications of childbirth) and O98 (Other unspecified maternal conditions). The statistical analysis was performed using SAS 9.4 software (SAS Institute Inc., Cary, NC, USA).

Pregnancies with no event were censored at the date of delivery or termination. Only pregnancies with start and end dates were used.

### Ethical and regulatory aspects

All patients included in the FHDH give informed consent for the use of their anonymized data, and for the publication of anonymized results from these data. This cohort has been approved by the Commission Nationale Informatique et Libertés (CNIL) since November 27th 1991, subsequently updated on March 30th 2021 (deliberation number 2021-044) and has led to several international publications.

## Results

Overall, there were 1,774 pregnancies in 881 women living with HIV. The mean age at the time of pregnancy was 30.3 years (SD = 6.6). Most pregnant women were from Haiti (39.2%), Suriname (26%), and France (15.4%), as in the overall cohort.

Table 1 shows HIV characteristics. Briefly, 69% of women had first been screened for HIV during pregnancy at the initiation of a physician. Only 14.9% of women had CD4 counts <200 per mm^3^ and 17% were at the Acquired Immuno Deficiency Syndrome (AIDS) stage. Between 1993 and 2002, the mean nadir CD4 count was 579 per mm^3^, between 2003 and 2012 it was 375 per mm^3^, and between 2013 and 2022 it was 509 per mm^3^.

**Table 1:** Description of HIV characteristics among 881 pregnant women living in French Guiana with details on associated STI and comorbidities.

Characteristics of HIV infection	Frequency
Hepatitis co-infection, *n*/*N* (%)	
Hepatitis B	15/881 (1.7%)
Hepatitis C	8/881 (0.9%)
Hepatitis B&C	1/881 (0.1%)
Mean age at HIV diagnosis (years) (SD^a^)	27.3 (7.2)
Mean duration of HIV infection (years since diagnosis) (SD)	11.6 (7.4)
Transmission mode, *n*/*N* (%)	
Heterosexual	830/881 (94.2%)
Unknown	31/881 (3.5%)
Materno-foetal	13/881 (1.5%)
Transfusion	1/881 (0.1%)
Accidental exposure to blood	1/881 (0.1%)
Hemophiliac/blood products	2/881 (0.2%)
Other	3/881 (0.3%)
Context of diagnosis, *N* (%)	
Pregnancy	216 (69.0%)
Screening without any notion of exposure to HIV	32 (10.2%)
HIV-related symptoms/clinical signs	28 (8.9%)
Risk of HIV exposure less than 6 months before	22 (7.0%)
Risk of exposure to HIV greater than 6 months	6 (1.9%)
Confirmation of known seropositivity	2 (0.6%)
Sexually transmitted infection	7 (2.2%)
Missing	568
CD4 lymphocytes at screening (per mm^3^), *N* (%)	
<200	87 (14.9%)
200–349	127 (21.8%)
350–500	139 (23.9%)
≥500	229 (39.3%)
Missing	299
HIV viral load at screening (copies/mm^3^), *N* (%)	
≤50	48 (9.0%)
51–1,000	71 (13.3%)
1,001–30,000	281 (52.7%)
>30,000	133 (25.0%)
Missing	348
Centers for Disease Control (CDC) stage, *N* (%)	
A	599 (68.1%)
B	131 (14.9%)
C	150 (17.0%)
Mean number of months between HIV diagnosis and AIDS (SD), (*N* = 150)	83.1 (81.9)
History of other sexually transmitted infection (STI), *n*/*N* (%)	44/881 (5.0%)
Types of STI, *n*/*N* (%)	
Chlamydia	5/881 (0.6%)
Neisseria gonorrhoea	
Ano-genital condylomas	12/881 (1.4%)
Herpes	14/881 (1.6%)
Syphilis	13/881 (1.5%)
Trichomoniasis	7/881 (0.8%)
Venereal vegetation	2/881 (0.2%)
History of opportunistic infection, *n*/*N* (%)	81/881 (9.2%)
Medical History	
Diabetes, *n*/*N* (%)	35/881 (4.0%)
Hypertension, *n*/*N* (%)	98/881 (11.1%)
Renal failure, *n*/*N* (%)	17/881 (1.9%)

^a^ SD, Standard deviation; AIDS, Acquired Immuno Depression Syndrome; HIV, Human Immunodeficiency Virus.

Table 2 shows that the HIV diagnosis was known in 75.1% of pregnancies and that, in 67.6% of women, HIV follow-up predated pregnancy. Nearly half of women, 49.4%, only had one pregnancy since having been diagnosed with HIV. Although most women received antiretroviral therapy during pregnancy, only 48.5% of those with the available information had an undetectable viral load at delivery. Between 2012 and 2022, 93% of pregnant women were on antiretroviral therapy.

**Table 2:** Description of characteristics and outcomes among 1,774 pregnancies among 881 women living with HIV: French Guiana 1992–2022.

Characteristics	Descriptive statistic^a^
Mean age at the time of pregnancy (SD) (years)	30.3 (6.6)
Start of pre-pregnancy follow-up, *N* (%)	1,199 (67.6%)
Number of pregnancies since HIV diagnosis, *N* (%)	
1	435 (49.4%)
2	202 (22.9%)
3	126 (14.3%)
>3	118 (13.4%)
Antiretroviral (ARV) treatment during pregnancy, *N* (%)	1,448 (81.6%)
Initiation of ARV treatment, *N* (%)	
During pregnancy	659 (45.5%)
Prior to pregnancy	789 (54.5%)
Number of ARV lines during pregnancy, *N* (%)	
1	1,006 (69.5%)
2	350 (24.2%)
3	74 (5.1%)
>3	18 (1.2%)
Viral load at delivery, copies per ml, *N* (%)	
≤ 50	666 (48.5%)
51–400	168 (12.2%)
401–1,000	83 (6.0%)
>1,000	457 (33.3%)
Missing	400
CD4 during pregnancy, per ml, *N* (%)	
< 200	189 (14.1%)
200–349	327 (24.4%)
350–499	337 (25.2%)
≥ 500	485 (36.2%)
Missing	436
Pregnancy outcome, *N* (%)	
Birth	1,481 (84.7%)
Abortion	267 (15.3%)
Preterm birth^b^	106/581 (18.2%)
Low birth weight among term births (<2,500 g)^c^	7/69 (17%)
Missing	26

^a^ *N = *1,774*.*

^b^ Cayenne maternity only.

^c^ Available birth weights in a Cayenne sample only.

Overall, 15.3% of pregnancies ended with an abortion but between 2012 and 2022 it significantly declined to 10.4%. Overall, 99.8% of women gave exclusive artificial feeding to their newborn. Since the beginning of data collection, there were a total of 110 newborns infected with HIV representing an overall transmission rate of 6.2% (110/1,771). Between 1993 and 2002, the transmission rate was 34%, between 2003 and 2012 it was 1.3%, and between 2013 and 2022 it was 0.7%.

**Figure 1:**
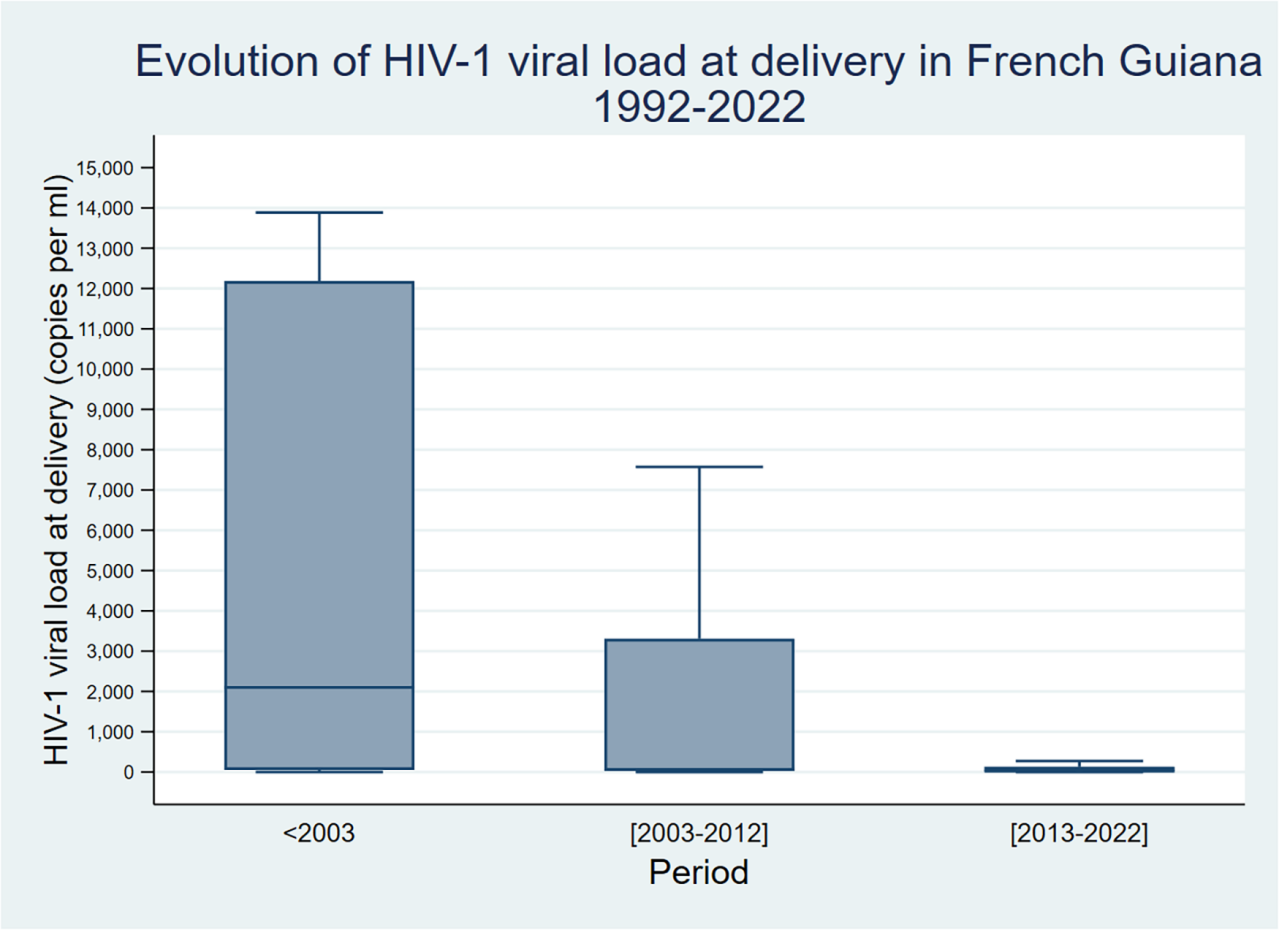
Evolution of HIV-1 viral load at delivery.

Figure 1 shows the gradual reduction of the viral load at delivery.

### Preterm deliveries

Overall, in Cayenne, since 2008, 106 of 581 (18.2%) pregnancies among women living with HIV with available information were premature before 37 weeks of pregnancy; of these, 33 (5.7%) were very preterm deliveries (28 to less than 32 weeks) and 73 (13.3%) were late preterm deliveries (<37 weeks and >32 weeks). In a sample from Cayenne maternity, the median birthweight was 3,050 g and 17% were considered to have low birth weight (<2,500 g). Among term births, 7/69 (10%) had low birth weight.

**Figure 2:**
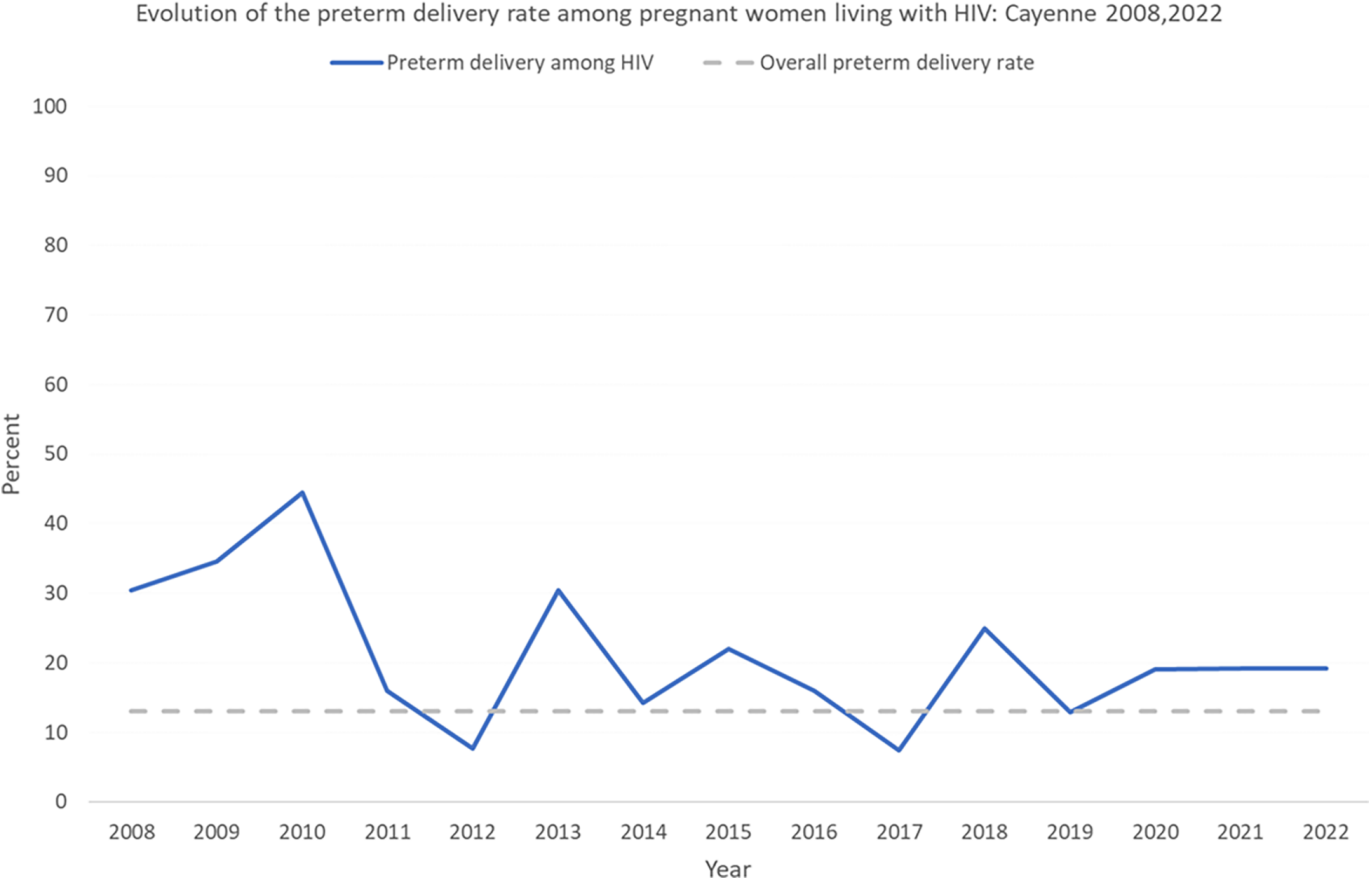
Evolution of the preterm delivery rate among pregnant women living with HIV in Cayenne.

Over time, in Cayenne, preterm delivery declined significantly (Figure 2, *χ*^2^ for trend *P* < 0. 019). Overall, 48 cases of preeclampsia (2.7%) were reported.

Supplementary Table S1 details the number of obstetrical events corresponding to the following ICD 10 codes: O10–O16, O20–O29, O40–O48, O60–O75 and O98.

## Discussion

Here, in a territory combining the highest HIV prevalence of all French territories, the highest fertility rate in Latin America, we provide the first epidemiologic overview of HIV-positive pregnancies. Among pregnant women living with HIV, we show that the preterm delivery rate remained particularly high: in 2022 it was 19.2% vs. 13% in the general population of French Guiana, and 6.9% in the general population of mainland France. The proportion of low birth weight births was also very high: 17% vs. 11.9% in the general population of French Guiana, and 7.5% in the general population of mainland France, and 14.6% in the world (14).

Over half (53%) of the general population of French Guiana lives below the poverty threshold, 29% lives in great poverty (15), and persons living with HIV are an even more precarious subpopulation. The link between poor follow-up, psychosocial stressors, and preterm delivery has been previously shown and presumably explain much of this difference (6). However, HIV itself may also contribute to this very high rate of preterm delivery (12). Other analytic studies in territories with widespread poverty have yielded contrasting conclusions: some did not demonstrate significant differences (13) whereas others did (12). Although the present study was only descriptive, the levels of preterm delivery or low birth weight were substantially greater than in the general population of French Guiana and it is likely that there are indeed worse pregnancy outcomes among women living with HIV than in the general female population of French Guiana. Given the progress in testing and treating women, we hypothesize that the remaining challenges regarding preterm delivery and low birth weight would most benefit from optimal obstetrical follow-up.

In French Guiana, health professionals and non-government organizations have been proactive in reaching vulnerable populations. These treatment and prevention efforts have now led to most women delivering with viral loads beneath transmission thresholds and very low residual transmission, usually in cases of women tested late in their pregnancy or lost to follow-up. Between 2012 and 2022, 93% of pregnant women had received ARV treatment, a figure that is only slightly above that of what is reported globally (13). This emphasizes the residual challenges that remain to follow the most vulnerable women, a fraction of whom escape the reach of the health system.

The present study has a number of limitations. The absence of a proper control group in the present study was a notable limitation. The crude comparisons relied on different data sources for the general population of French Guiana. Our analysis was purely descriptive and spans over 3 decades of multiple gradual changes, in terms of social conditions, of HIV care and more generally of the organization of the health system. It is therefore difficult to pinpoint any single factor involved in the positive trends but rather a convergence of proactive efforts to reach and test pregnant women, improvements of the coordination of health care workers, improvement in treatment strategies, improvements in the tolerance and effectiveness of antiretrovirals being a potent combination to reduce vertical transmission of HIV. Another important limitation is that social factors are probably the major determinant of the studied health outcomes, yet we had no direct measurement of precariousness in our data, so we can only assume –not prove—that much of what is shown is detrimentally influenced by precariousness. The information about preterm delivery rests on a subsample from Cayenne's level 3 maternity which may bias preterm delivery estimates upwards. Furthermore, the data on birthweight was also a subsample from Cayenne maternity so results should be taken with caution. Despite the above limitations, the present study has some merits as it is the first study to provide real life data on historical trends and remaining challenges in French Guiana; it also provides important information on various standard aspects of HIV infection and their temporal evolution.

In summary, the present study shows that with time the system has been successful in reducing mother to child transmission and preterm births. However, challenges remain because incidence of preterm delivery and low birth weight are still substantially higher than in the general population of pregnant women in French Guiana. Teasing out what fraction is linked to HIV and what fraction is linked to social precariousness and poor follow-up was not possible in this study but, in practice, it seems crucial to implement to continue early and careful obstetrical follow-up for pregnant women living with HIV. Reducing maternal-fetal transmission of HIV necessarily implies efforts to improve access to care for fragile populations.

## Data Availability

The data analyzed in this study is subject to the following licenses/restrictions. According to French Law data cannot be sent to other structures without prior authorization by the Commission Nationale Informatique et Libertés. Upon resonable request and with permission from the CNIL anonymized data may be shared. Requests to access these datasets should be directed to corevih@ch-cayenne.fr.
