# Supplementary material for: Thirty years of HIV pregnancies in French Guiana: prevention successes and remaining obstetrical challenges
Source: Front Glob Womens Health. 2024 Jan 3;4:1264837. doi: 10.3389/fgwh.2023.1264837 (PMC10791775; doi:10.3389/fgwh.2023.1264837)
Supplement: Supplementary file 1 [file Table1.docx]

**Supplementary table 1. Obstetrical events during pregnancy.**

| **Events :** |  |
| --- | --- |
| Other maternal conditions mainly related to pregnancy | 63 (3.6%) |
| Other maternal illnesses classifiable elsewhere but complicating pregnancy, childbirth and the puerperium | 16 (0.9%) |
| Labor and delivery complications | 129 (7.3%) |
| Obstetrical complications | 112 (6.3%) |
| Complications mainly related to puerperium | 21 (1.2%) |
| Obstetrical deaths | 2 (0.1%) |
| Infectious and parasitic diseases of the mother classified elsewhere but complicating pregnancy, childbirth and the puerperium | 17 (1%) |
| Maternal care related to the fetus and amniotic cavity | 26 (1.5%) |
| Edema, proteinuria and hypertension in pregnancy, childbirth and the puerperium | 49 (2.8%) |
|  |  |
| **Event details :** |  |
| **Edema, proteinuria and hypertension in pregnancy, childbirth and the puerperium:** |  |
| Eclampsia sai | 1 (0.1%) |
| Hypertension related to pre-existing nephropathy, complicating pregnancy, childbirth and the puerperal period | 1 (0.1%) |
| Maternal hypertension, unspecified | 2 (0.1%) |
| Pre-existing essential hypertension complicating pregnancy, childbirth and the puerperium | 7 (0.4%) |
| Gestational [pregnancy-related] hypertension (without significant proteinuria) | 7 (0.4%) |
| Gestational hypertension sai | 8 (0.5%) |
| Severe preeclampsia | 1 (0.1%) |
| Moderate preeclampsia | 5 (0.3%) |
| Severe preeclampsia | 3 (0.2%) |
| Preeclampsia, unspecified | 13 (0.7%) |
| Eclampsia in pregnancy | 6 (0.3%) |
| Eclampsia in labor | 1 (0.1%) |
| Eclampsia, period not specified | 1 (0.1%) |
|  |  |
| **Other maternal conditions mainly related to pregnancy:** |  |
| Pregnancy-related condition, unspecified | 1 (0.1%) |
| Liver disorders during pregnancy, childbirth, and the puerperium | 1 (0.1%) |
| Other specified pregnancy-related conditions | 1 (0.1%) |
| Other vomiting complicating pregnancy | 1 (0.1%) |
| Diabetes and sugar during pregnancy, childbirth, and the puerperium | 1 (0.1%) |
| Diabete sucre gestationnel sai | 3 (0.2%) |
| Diabetes mellitus in pregnancy | 4 (0.2%) |
| Diabetes mellitus during pregnancy, unspecified | 11 (0.6%) |
| Diabetes mellitus during pregnancy | 26 (1.5%) |
| Hyperemesis gravidarum, benign or without precision, beginning before the end of the 22nd week of gestation | 1 (0.1%) |
| Hyperemesis gravidarum with metabolic disorders | 1 (0.1%) |
| Benign hyperemesis gravidarum | 1 (0.1%) |
| Hemorrhoids in pregnancy | 1 (0.1%) |
| Urinary tract infection in pregnancy | 2 (0.1%) |
| Genital tract infections in pregnancy | 2 (0.1%) |
| Renal infections in pregnancy | 3 (0.2%) |
| Threat of abortion | 7 (0.4%) |
| Excessive weight gain during pregnancy | 1 (0.1%) |
| Abnormal result found during the mother's routine prenatal examination, unspecified | 1 (0.1%) |
| Thrombophlebitis (superficial) in pregnancy | 1 (0.1%) |
| Vomiting in pregnancy, unspecified | 1 (0.1%) |
|  |  |
| **Obstetrical complications :** |  |
| Abnormal amniotic fluid and membranes, unspecified | 1 (0.1%) |
| Placenta anomaly, unspecified | 2 (0.1%) |
| Other amniotic fluid and membrane anomalies | 2 (0.1%) |
| Other placental anomalies | 1 (0.1%) |
| Other specified abnormalities of amniotic fluid and membranes | 3 (0.2%) |
| Other premature abruptions of the placenta | 3 (0.2%) |
| Premature placental abruption, unspecified | 1 (0.1%) |
| False labor before 37 full weeks of gestation | 26 (1.5%) |
| False work, without precision | 26 (1.5%) |
| Prolonged pregnancy | 8 (0.5%) |
| Retroplacental hematoma with hemorrhage (significant) associated with disseminated intravascular coagulation | 1 (0.1%) |
| Retroplacental hematoma sai | 5 (0.3%) |
| Hydramnios | 3 (0.2%) |
| Pre-delivery hemorrhage, unspecified | 1 (0.1%) |
| Birth after term | 1 (0.1%) |
| Oligoamnios | 2 (0.1%) |
| Precise placenta previa without hemorrhage | 1 (0.1%) |
| Premature rupture of the membranes | 2 (0.1%) |
| Premature rupture of membranes, with onset of labor beyond 24 hours | 11 (0.6%) |
| Premature rupture of membranes, with onset of labor within 24 hours | 16 (0.9%) |
| Premature rupture of membranes, unspecified | 7 (0.4%) |
| Premature rupture of membranes, labor delayed by treatment | 1 (0.1%) |
| Placental transfusion syndromes | 1 (0.1%) |
|  |  |
| **Labor and delivery complications :** |  |
| Uterine atony | 1 (0.1%) |
| Other immediate postpartum hemorrhages | 1 (0.1%) |
| Other specified obstetric injuries | 1 (0.1%) |
| Insufficient initial (uterine) contractions | 5 (0.3%) |
| Hypertonic, uncoordinated, and prolonged uterine contractions | 1 (0.1%) |
| (Spontaneous) onset of labour before 37 full weeks of gestation | 12 (0.7%) |
| Insufficient cervical dilatation | 1 (0.1%) |
| Dystocia due to fetopelvic disproportion, unspecified | 1 (0.1%) |
| Dystocia due to face presentation | 1 (0.1%) |
| Dystocia due to breech presentation | 3 (0.2%) |
| Twin dystocia | 1 (0.1%) |
| Dystocia, unspecified | 1 (0.1%) |
| Obstetric cervical tear | 1 (0.1%) |
| Obstetrical tear of the perineum | 1 (0.1%) |
| Second-degree obstetric tear of the perineum | 1 (0.1%) |
| First-degree obstetric tear of the perineum | 46 (2.6%) |
| Third-degree obstetric tear of the perineum | 1 (0.1%) |
| Obstetrical tear of the perineum, unspecified | 6 (0.3%) |
| Isolated high vaginal obstetric tear | 1 (0.1%) |
| Postpartum hemorrhage (uterine atony) sai | 1 (0.1%) |
| Postpartum hemorrhage due to retention of products of conception after delivery | 1 (0.1%) |
| Uterine hypertonia | 1 (0.1%) |
| Delivery hemorrhage (third period) | 6 (0.3%) |
| Late and secondary postpartum hemorrhage | 2 (0.1%) |
| Overtime of the second period [expulsion]. | 2 (0.1%) |
| Extending the first period [dilation]. | 1 (0.1%) |
| Pyrexia in labour, not elsewhere classified | 2 (0.1%) |
| Rupture of the uterus during labor | 1 (0.1%) |
| Retention of placenta without hemorrhage | 2 (0.1%) |
| Partial retention of placenta and membranes, without hemorrhage | 1 (0.1%) |
| Labour and delivery complicated by other umbilical cord anomalies | 1 (0.1%) |
| Labour and delivery complicated by cord prolapse | 1 (0.1%) |
| Labour and delivery complicated by abnormal fetal heart rate | 17 (1%) |
| Labour and delivery complicated by abnormal fetal heart rate with meconium in amniotic fluid | 1 (0.1%) |
| Labour and delivery complicated by abnormal fetal heart rate with meconium in amniotic fluid | 6 (0.3%) |
| Labour and delivery complicated by another form of cord entanglement | 1 (0.1%) |
| Labour and delivery complicated by cord circularity, with compression | 2 (0.1%) |
| Labour and delivery complicated by fetal distress, unspecified | 1 (0.1%) |
| Labor and delivery complicated by meconium in amniotic fluid | 20 (1.1%) |
| Working too fast | 6 (0.3%) |
| Failed vacuum and forceps application, without precision | 1 (0.1%) |
| Failure to induce labor | 1 (0.1%) |
|  |  |
| **Complications mainly related to puerperium:** |  |
| Other puerperal complications, not elsewhere classified | 3 (0.2%) |
| Other venous complications during puerperium | 1 (0.1%) |
| Other genital tract infections after childbirth | 1 (0.1%) |
| Puerperal complication, unspecified | 1 (0.1%) |
| Obstetrical pulmonary embolism sai (due to blood clot) | 1 (0.1%) |
| Puerperal endometritis | 1 (0.1%) |
| Galactorrhea | 1 (0.1%) |
| Obstetric wound hematoma | 1 (0.1%) |
| Infection of an obstetric surgical wound | 5 (0.3%) |
| Suture infection after cesarean section | 2 (0.1%) |
| Urinary tract infection after childbirth | 1 (0.1%) |
| Puerperal sepsis | 1 (0.1%) |
| Suppression of lactation | 5 (0.3%) |
|  |  |
| **Infectious and parasitic diseases of the mother classified elsewhere but complicating pregnancy, childbirth and the puerperium:** |  |
| Other infectious and parasitic diseases of the mother complicating pregnancy, childbirth and the puerperium | 1 (0.1%) |
| Other viral diseases complicating pregnancy, childbirth and the puerperium | 7 (0.4%) |
| Disease caused by the human immunodeficiency virus [HIV], complicating pregnancy, childbirth and the puerperium. | 9 (0.5%) |
